# Supplementary material for: Spatial Risk Distribution of Lumpy Skin Disease in Thailand Based on Maximum-Entropy Modeling
Source: Animals (Basel). 2025 Aug 21;15(16):2456. doi: 10.3390/ani15162456 (PMC12382764; doi:10.3390/ani15162456)
Supplement: Supplementary file 1 [file animals-15-02456-s001.zip › animals-3806879-supplementary.pdf]

Supplementary Materials

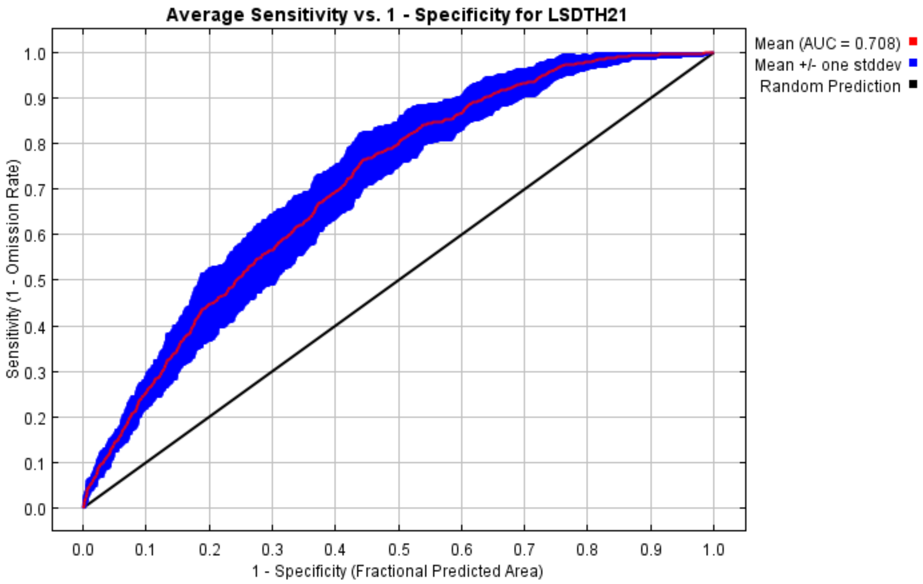

Supplement Figure S1. MaxEnt receiver operating characteristic curve for lumpy skin disease model using LSD 2021 dataset. The mean area under the curve of 0.708 reflects moderate model performance, and the shaded area represents variability across runs.

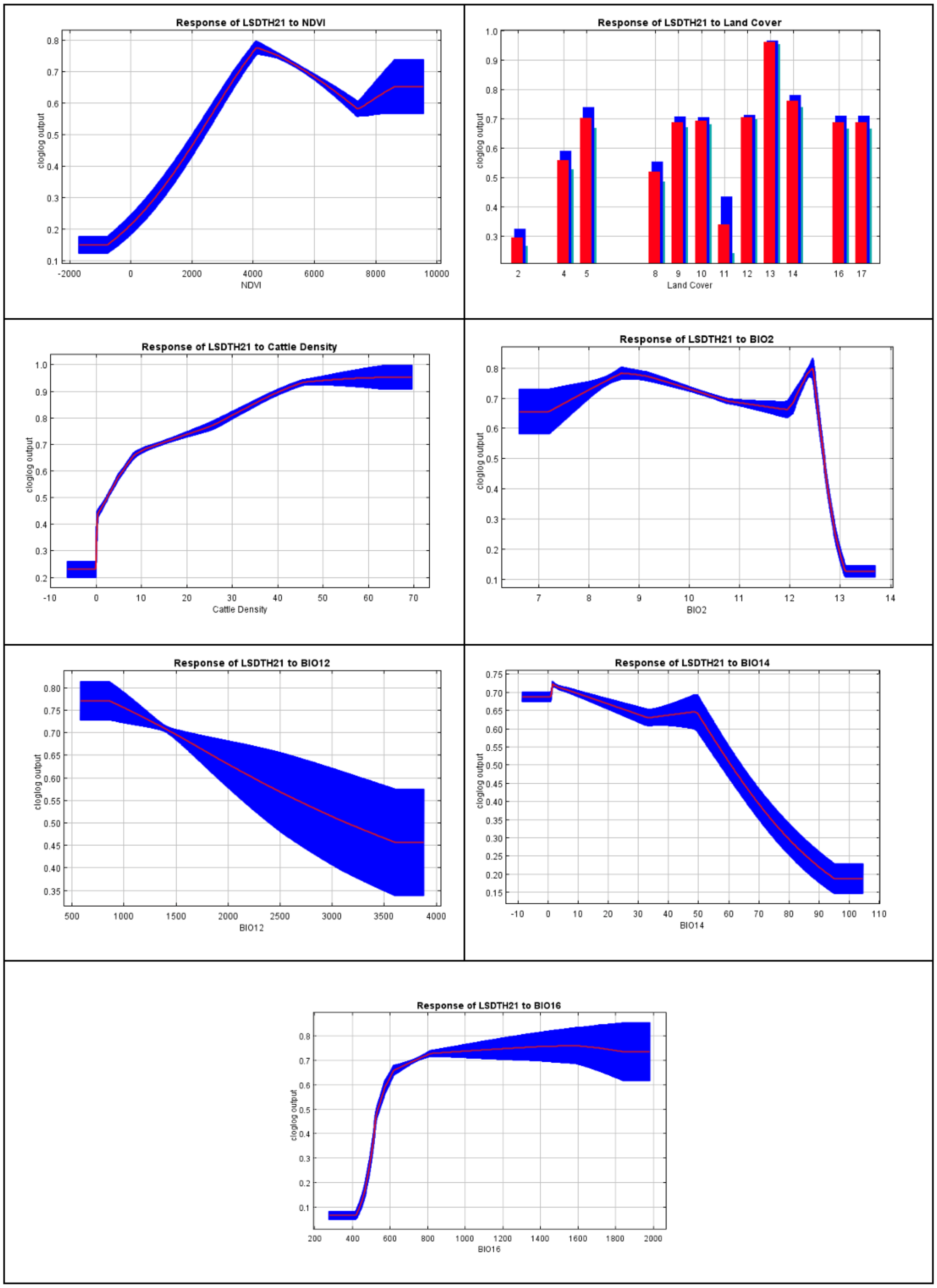

Supplement Figure S2. Response curves of environmental variables influencing lumpy skin disease model using LSD 2021 dataset. The MaxEnt model shows the effect of each variable on disease suitability. Red lines indicate mean responses; blue areas represent standard deviation.

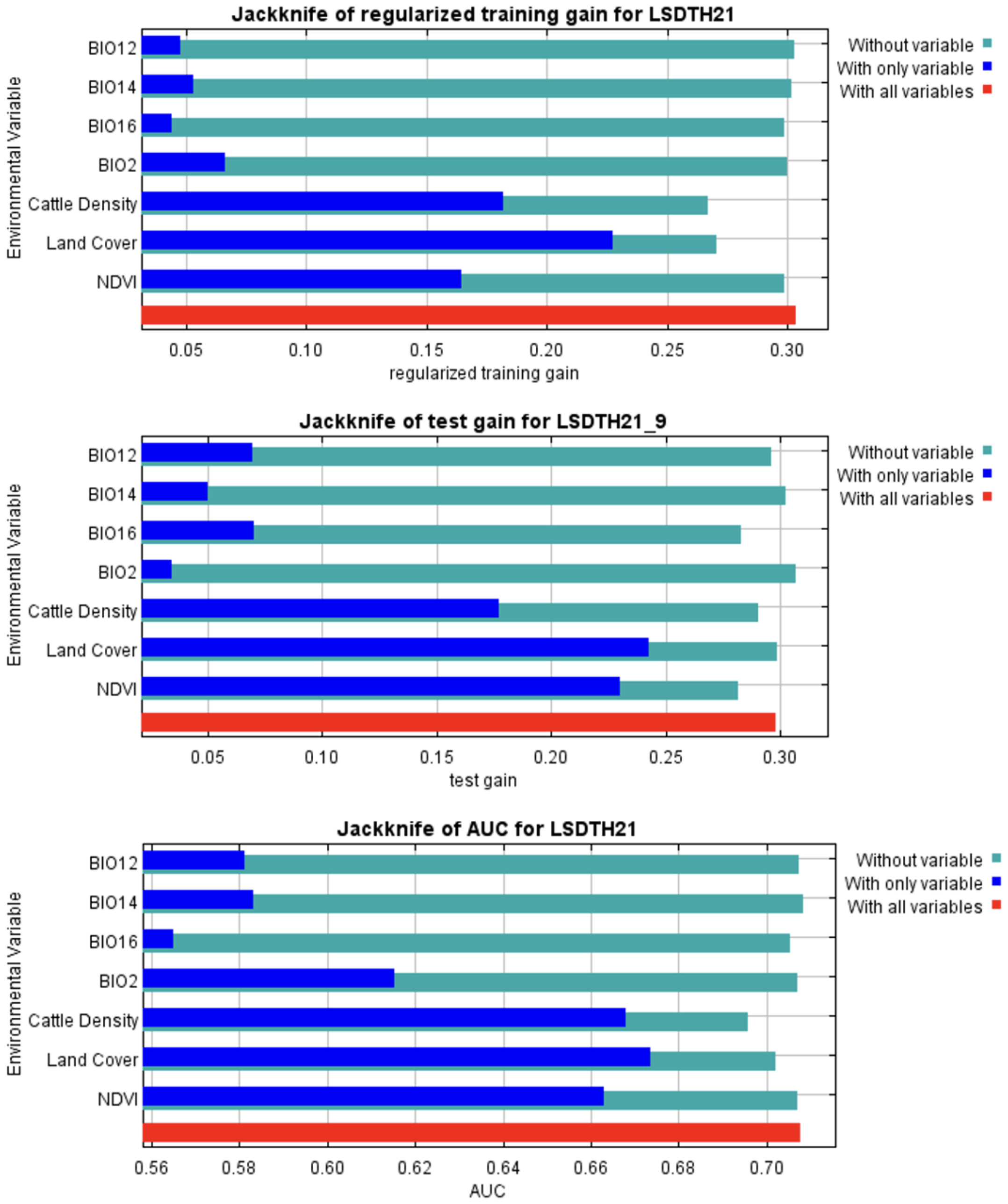

Supplement Figure S3. The jackknife test for evaluating the relative importance of environmental variables using LSD 2021 dataset. Land cover contributed the most to model, followed by cattle density and NDVI, while bioclimatic variables showed limited influence.
